# Molecular quantum robotics: particle and wave solutions, illustrated by “leg-over-leg” walking along microtubules

**DOI:** 10.3389/fnbot.2015.00002

**Published:** 2015-05-08

**Authors:** Paul Levi

**Affiliations:** Forschungszentrum Informatik (Centre of Computer Science), Intelligent System and Production Engineering, Interactive Diagnosis and Service SystemsKarlsruhe, Germany

**Keywords:** molecular robotics, biped walking on microtubules, motion of kinesin-1 and dynein alog axons and dendrites, particles and wave solutions, neuro-robotics

## Abstract

Remarkable biological examples of molecular robots are the proteins kinesin-1 and dynein, which move and transport cargo down microtubule “highways,” e.g., of the axon, to final nerve nodes or along dendrites. They convert the energy of ATP hydrolysis into mechanical forces and can thereby push them forwards or backwards step by step. Such mechano-chemical cycles that generate conformal changes are essential for transport on all different types of substrate lanes. The step length of an individual molecular robot is a matter of nanometers but the dynamics of each individual step cannot be predicted with certainty (as it is a random process). Hence, our proposal is to involve the methods of quantum field theory (QFT) to describe an overall reliable, multi–robot system that is composed of a huge set of unreliable, local elements. The methods of QFT deliver techniques that are also computationally demanding to synchronize the motion of these molecular robots on one substrate lane as well as across lanes. Three different challenging types of solutions are elaborated. The impact solution reflects the particle point of view; the two remaining solutions are wave based. The second solution outlines coherent robot motions on different lanes. The third solution describes running waves. Experimental investigations are needed to clarify under which biological conditions such different solutions occur. Moreover, such a nano-chemical system can be stimulated by external signals, and this opens a new, hybrid approach to analyze and control the combined system of robots and microtubules externally. Such a method offers the chance to detect mal-functions of the biological system.

## Introduction

Molecular robotics, which operates on a nano scale, has in the last decade witnessed impressive growth, (e.g., Murata et al., 2013). The current topics in this field are molecular machines (Balzani et al., 2008; Roux, 2011; Fukuda et al., 2012; Seeman, 2014) and, even more important in the context of this contribution, biped DNA walkers (Sherman and Seeman, 2004; Shin and Pierce, 2004; Omabegho et al., 2009; Lund et al., 2010). In a previous contribution, the biological process of muscle contraction by the “one-legged” motion of myosin II along actin-filaments has been described using QFT methods (Haken and Levi, 2012). The results of different types of synchronization of multi-molecular systems have been conclusive in the sense that beside the more classical impact solution, two quantum mechanical solutions exist that can describe the synchronization of billions of unreliable molecules. This result can usually not be modeled by pure classical particle solutions. Despite the theoretical existence of these innovative solutions, knowledge of a great set of experimentally confirmed data is unfortunately still now very limited. Nevertheless, these theoretical outcomes encourage us to continue with our quantum theoretical approach, since we are convinced that in the near future confirming experimental data will be available.

This paper focuses on a description of the “leg-over-leg” motion of molecular robots along tubulin strands. These biological nano-robots (motor proteins) walk along such lanes in a four-step process, and are fueled by ATP consumption (in physics, energy exchange with a heat bath) after the first and third steps. From a system perspective, this denotes that we are modeling an open system that is not in a thermal equilibrium. The C-terminal domain, e.g., of kinesin-1, act as a gripper and is attached to cargo, e.g., vesicles that are transported along axons and dendrites, which are both connected to neuron cell bodies. Very similar processes occur for the much larger dynein, with two legs (in biological terms two “heads”), which also transports vesicles along axons and dendrites. Due to this similarity, this paper focuses on a general description of the motion processes of both molecular robots. We abandon the modeling of cargo transport in this paper for the sake of understandability.

In this paper, a set of such molecules is regarded as a swarm of molecular robots that must be configured, synchronized, and in real time supported by energy to perform the next step (Levi and Haken, 2010). Due to the utilization of a quantum theoretical approach, such molecules can be described as particles or as waves (according to matter/field dualism). The following three different solutions are presented:

1. Impact (stroke) solution of one molecular robot on a single substrate lane,
2. Coherent motion of many robots on and between parallel substrate lanes,
3. Running wave that synchronizes the motions of many robots on one substrate lane.

In all three solutions, a local *B*-field is activated within a lane and across different lanes. Such a field not only controls the process of energy consumption but also, even more importantly, acts as a signal field that synchronizes the steps of the molecular robots. This field is generated by the molecular robots themselves and is not externally injected.

The impact solution pertains to the motion of a single molecular robot on one lane. Damping effects transform the typical wave characteristics of a QFT approach to particle behavior. The appearance of a sequence of impacts pushes the molecule (particle) forwards or backwards step by step.

The next two solutions plainly reveal the wave features of our approach. The coherent motion solution distinguishes between the individual robots walking on a lane and across the lanes. In contrast to the second solution, the running wave solution presents a result that can be obtained in special restrictive conditions.

## The model

The “leg-over-leg” walking of a kinesin molecule (dynein) is modeled as a bipedal molecular robot *r*, where the two heads (light chains) are considered as two legs, and the “coiled-coil tail” as an effector (Alberts et al., 2008). The track is a microtubule surface (substrate lane), along which *r* moves in discrete steps. During such a walk, a robot *r* can take one of four leg states and one of two walking states:

                *Leg state* a:  leg 1 or leg 2 points backwards.
                *Leg state* b:  leg 1 or leg 2 points forwards.
*Leg-over-leg state c*_1_:  leg 1 is loosely bound to the top of leg 2, which is tightly attached to the substrate.
*Leg-over-leg state* c_2_:  leg 2 is loosely bound to the top of leg 1, which is tightly attached to the substrate.
         *Walking state* 1:  *r* is moving forward with leg 1.
         *Walking state* 2:  *r* is moving forward with leg 2.

**Figure 1:**
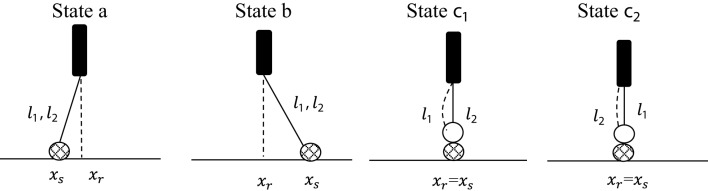
**Representation of the two *leg states* a and b and the two *leg-over-leg* states c_1_ and c_2_**. The walking direction is from left to right. For simplicity, we present in this diagram each leg as tightly connected to the substrate (crossed circle). The loosely bound states connected to the substrate are omitted (see Figure 2).

Figure 1 shows the four different leg states.

The two legs are attached to the substrate in two different connection states:

*Connection state* 1: leg 1 is tightly attached to position *p*_*k*_ of the tubulin substrate and leg 2 is weakly attached to site *p*_*k* + 1_.
*Connection state* 2: leg 2 is tightly attached to site *p*_*k*_ and leg 1 is weakly attached to position *p*_*k* + 1_ of the substrate.

The level of attachment to the substrate in these two connection modes is defined by the two possible states of the substrate:

*Ground state* g: represents a weak attachment of a leg to the substrate molecule.
*Excited state* e: represents a strong attachment of a leg to the substrate molecule.

**Figure 2:**
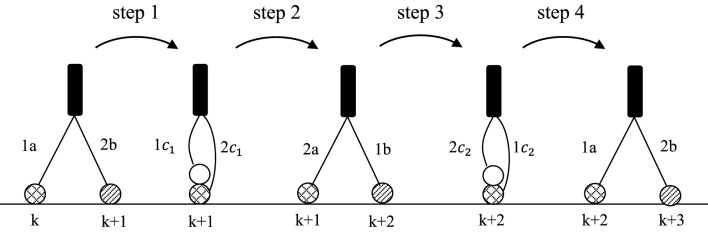
**Demonstration of one complete “leg-over-leg” cycle of a molecular robot *r* which walks along a substrate lane**. The loosely bound states are marked by crossed circles 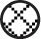. The tightly bound states are marked by striped circles 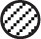. The first position is fixed by *k* = 1; the initial state is (1a, 2b).

The discrete periodic movement of the molecular robot *r* is characterized by a four-step cycle (Figure 2) that starts with the two leg states (1a, 2b), continues with the transference of these states in step 1 into the leg-over-leg state (1c_1_, 2c_1_), and is maintained by step 2, which produces the leg states (2a, 1b). In step 3, the leg-over-leg state (1c_2_, 2c_2_) is achieved, and finally, in step 4, the states (1a, 2b) are again established.

The following notation is employed for the creation (or annihilation) operators:

*Molecular robot r*: *r*^†^_*leg*_1__ _*state*_*j*__ _*position*_*k*_;_ _*leg*_2__ _*state*_*j*′__ _*position*_*k*′_;_ e.g., *r*^†^_*l*_1*ap*1_;_ _*l*_2_*bp*_2__.
*Substrate s*: *s*^†^_*state*_*j*___*position*_*k*__; e.g., *s*^†^_*gp*__1_.

Table 1 subsumes the different individual motion patterns of the two legs of *r*.

**Table 1:**
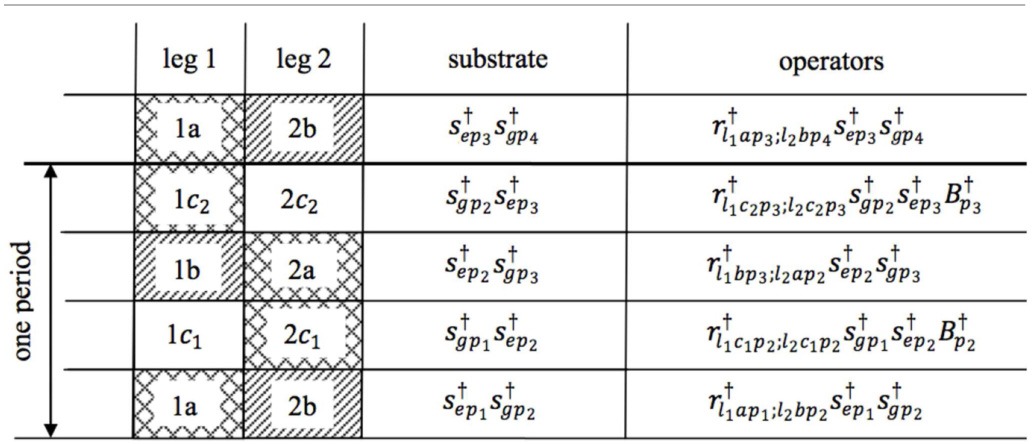
**Representation of the motion patterns of a “leg-over-leg” walking process of a molecular robot *r***.

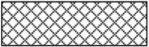
: tightly bound; 
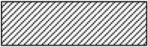
: looseley bound; 
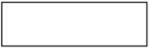
: leg up.

The initial state is (1a, 2b).

The energy transfer process (*s* → *r*) occurs in steps 1 and 3 and is combined with the excited status of the substrate. In addition, the heat-bath operators (“fueling” and synchronization operators) *B*^†^_*p*__*k*_ and *B*^†^_*p*__*k*_ are applied in this walking phase. The operator transfers during the four steps are summarized as follows (readout from Table 1):

**Table:** 

initial state	step 1	“leg-over-leg” state
$r_{l_{1}a\;p_{1};\;l_{2}\;b\;p_{2}}^{†}s_{e\;p_{1}}^{†}s_{g\;p_{2}}^{†}$	→	$r_{l_{1}c_{1}\;p_{2};\;l_{2}\;c_{1}\;p_{2}}^{†}s_{g\;p_{1}}^{†}s_{e\;p_{2}}^{†}B_{p_{2}}^{†}$.
“leg-over-leg” state	step 2	reversed initial state
$r_{l_{1}c_{1}p_{2};\;l_{2}c_{1}p_{2}}^{†}s_{gp_{1}}^{†}s_{ep_{2}}^{†}B_{p_{2}}^{†}$	→	$r_{l_{1}b\;p_{3};\;l_{2}ap_{2}}^{†}s_{e\;p_{2}}^{†}s_{gp_{3}}^{†}$.
reversed initial state	step 3	“leg-over-leg” state
$r_{l_{1}bp_{3};\;l_{2}ap_{2}}^{†}s_{ep_{2}}^{†}s_{gp_{3}}^{†}$	→	$r_{l_{1}c_{2}\;p_{3};\;l_{2}\;c_{2}p_{3}}^{†}s_{gp_{2}}^{†}s_{ep_{3}}^{†}B_{p_{3}}^{†}$.
“leg-over-leg” state	step 4	initial state
$r_{l_{1}c_{2}p_{3};\;l_{2}c_{2}p_{3}}^{†}s_{gp_{2}}^{†}s_{ep_{3}}^{†}B_{p_{3}}^{†}$	→	$r_{l_{1}a\;p_{3};\;l_{2}bp_{4}}^{†}s_{e\;p_{3}}^{†}s_{g\;p_{4}}^{†}$.

This transfer list of operators delivers the definition of the interaction Hamiltonian *H*_*int*_ and finally the elaboration of the resulting equations of motion.

## Interaction hamiltonian

The local interaction Hamiltonian *H*_*int*_ at positions to *p*_1_ to *p*_4_ is defined by expression 3.1, where the first step starts at the initial site *k* marked in Figure 2. Further, two real coupling constants, *g*_1_ and *g*_2_, are introduced, where the parameter *g*_1_ describes the uneven steps and *g*_2_ denotes the even steps.

(3.1) $$\begin{array}{l} H_{int}=\;\hbar g_{1}r_{l_{1}a\;p_{1};\;l_{2}\;b\;p_{2}}^{†}s_{e\;p_{1}}^{†}s_{g\;p_{2}}^{†}r_{l_{1}c_{1}\;p_{2};\;l_{2}\;c_{1}p_{2}}s_{g\;p_{1}}s_{e\;p_{2}}\;B_{p_{2}}\\ \;+\hbar g_{1}s_{g\;p_{1}}^{†}s_{e\;p_{2}}^{†}r_{l_{1}c_{1}\;p_{2};\;l_{2}\;c_{1}\;p_{2}}^{†}\;s_{e\;p_{1}}s_{g\;p_{2}}r_{l_{1}a\;p_{1}\;;\;l_{2}\;b\;p_{2}}B_{p_{2}}^{†}\\ \;+\hbar g_{2}r_{l_{1}c_{1}\;p_{2};\;l_{2}\;c_{1}\;p_{2}}^{†}s_{g\;p_{1}}^{†}s_{g\;p_{3}}r_{l_{1}b\;p_{3};\;l_{2}\;a\;p_{2}}B_{p_{2}}^{†}\\ \;+\hbar g_{2}r_{l_{1}b\;p_{3};\;l_{2}\;a\;p_{2}}^{†}s_{g\;p_{3}}^{†}s_{g\;p_{1}}r_{l_{1}c_{1}\;p_{2};\;l_{2}\;c_{1}p_{2}}B_{p_{2}}\\ \;+\hbar g_{1}r_{l_{1}b\;p_{3};\;l_{2}\;a\;p_{2}}^{†}s_{g\;p_{2}}s_{\;e\;p_{2}}^{†}s_{e\;p_{3}}s_{\;g\;p_{3}}^{†}r_{l_{1}c_{2}\;p_{3};\;l_{2}\;c_{2}\;p_{3}}\;B_{p_{3}}\\ \;+\hbar g_{1}r_{l_{1}c_{2}\;p_{3};\;l_{2}\;c_{2}\;p_{3}}^{†}\;s_{e\;p_{2}}s_{g\;p_{2}}^{†}s_{g\;p_{3}}s_{e\;p_{3}}^{†}r_{l_{1}b\;p_{3}\;;\;l_{2}\;a\;p_{2}}\;B_{p_{3}}^{†}\\ \;+\hbar g_{2}r_{l_{1}c_{2}\;p_{3};\;l_{2}\;c_{2}\;p_{3}}^{†}s_{g\;p_{2}}^{†}s_{g\;p_{4}}r_{l_{1}a\;p_{3};\;l_{2}\;b\;p_{4}}B_{p_{3}}^{†}\\ \;+\hbar g_{2}r_{l_{1}a\;p_{3};\;l_{2}\;b\;p_{4}}^{†}s_{g\;p_{4}}^{†}s_{g\;p_{2}}r_{l_{1}c_{2}\;p_{3};\;l_{2}\;c_{2}\;p_{3}}B_{p_{3}}. \end{array}$$

By the replacements *p*_1_ → *p*_*k*_, *p*_2_ → *p*_*k* + 1_, etc., *H*_*int*_ is transferred into *H*_*int*, *k*_ and has to be summed up by *k* = 1, 2, …. All operators that are quoted here are Bose operators. Generally, we should involve both Fermi operators (anti-commutation rule) and Bose operators (commutation rule) for the definition of *H*_*int*_ and the resulting calculations of the Heisenberg equations of motions. Here, we use only Bose operators because in this case aggregations of molecules are allowed since the Pauli Exclusion Principle does not have to be applied. We neglect Fermi operators that depict supplementary interactions of Fermions with Bosons.

Endowed with the Hamiltonian *H*_*int*_, the corresponding full equations of motion have been calculated and are presented in the next sub-chapter.

## Full heisenberg equations of motion

The full set of Heisenberg equations of motion is completed by 7 damping constants, γ_*ab*_, etc., 7 fluctuating forces, *F*^†^_*ab*_, etc., and two coupling constants, *g*_1_, *g*_2_, and is defined by the following expressions, representing coupled nonlinear, delayed, and complex operator equations.

(4.1) $$\begin{array}{l} {\dot{r}}_{l_{1}ap_{k};l_{2}bp_{k+1}}^{†}=\;ig_{1}\left[s_{ep_{k}}s_{gp_{k}}^{†}r_{l_{1}c_{1}\;p_{k+1};l_{2}\;c_{1}p_{k+1}}^{†}s_{gp_{k+1}}s_{ep_{k+1}}^{†}\;B_{p_{k+1}}^{†}\right]\\ \;+ig_{2}\left[s_{gp_{k-1}}^{†}s_{gp_{k+1}}r_{l_{1}c_{2}\;p_{k};\;l_{2}\;c_{2}\;p_{k}}^{†}B_{\;p_{k}}^{†}\right]\\ \;-\gamma_{ab}r_{l_{1}ap_{k};\;l_{2}bp_{k+1}}^{†}+\;F_{ab}^{†}. \end{array}$$

(4.2) $$\begin{array}{l} {\dot{r}}_{l_{1}bp_{k+1};\;l_{2}ap_{k}}^{†}=\;ig_{1}\left[s_{e\;p_{k}}s_{g\;p_{k}}^{†}r_{l_{1}c_{2}\;p_{k+1};\;l_{2}c_{2}p_{k+1}}^{†}s_{gp_{k+1}}s_{ep_{k+1}}^{†}B_{\;p_{k+1}}^{†}\right]\\ \;+ig_{2}\;\left[s_{gp_{k-1}}^{†}s_{g\;p_{k+1}}\;r_{l_{1}c_{1}\;p_{k};\;l_{2}\;c_{1}\;p_{k}}^{†}B_{\;p_{k}}^{†}\right]\\ \;-\gamma_{ba}r_{l_{1}b\;p_{k+1};\;l_{2}ap_{k}}^{†}+\;F_{ab}^{†}. \end{array}$$

(4.3) $$\begin{array}{l} {\dot{r}}_{l_{1}c_{1}\;p_{k};\;l_{2}\;c_{1}\;p_{k}}^{†}=\;ig_{1}\left[s_{ep_{k-1}}^{†}s_{gp_{k-1}}r_{l_{1}ap_{k-1};\;l_{2}\;b\;p_{k}}^{†}s_{gp_{k}}^{†}s_{e\;p_{k}}B_{\;p_{k}}\right]\\ \;+ig_{2}\left[s_{gp_{k-1}}s_{gp_{k+1}}^{†}r_{l_{1}b\;p_{k+1};\;l_{2}a\;p_{k}}^{†}B_{\;p_{k}}\right]\\ \;+\gamma_{c_{1}}r_{l_{1}c_{1}p_{k};\;l_{2}c_{1}p_{k}}^{†}+\;F_{c_{1}}^{†}. \end{array}$$

(4.4) $$\begin{array}{l} {\dot{r}}_{l_{1}c_{2}\;p_{k};\;l_{2}\;c_{2}\;p_{k}}^{†}=\;ig_{1}\left[s_{gp_{k-1}}s_{ep_{k-1}}^{†}r_{l_{1}b\;p_{k};\;l_{2}\;a\;p_{k-1}}^{†}s_{ep_{k}}s_{gp_{k}}^{†}B_{\;p_{k}}\right]\\ \;+ig_{2}\left[r_{l_{1}a\;p_{k};\;l_{2}\;b\;p_{k+1}}^{†}s_{gp_{k-1}}^{†}s_{gp_{k+1}}B_{\;p_{k}}\right]\\ \;+\gamma_{c_{2}}r_{l_{1}c_{2}\;p_{k};\;l_{2}\;c_{2}p_{k}}^{†}+\;F_{c_{2}}^{†}. \end{array}$$

(4.5) $$\begin{array}{l} {\dot{s}}_{gp_{k}}^{†}=\;ig_{1}[r_{l_{1}a\;p_{k};\;l_{2}\;b\;p_{k+1}}^{†}\;s_{e\;p_{k}}^{†}s_{g\;p_{k+1}}^{†}s_{ep_{k+1}}r_{l_{1}\;c_{1}\;p_{k+1};\;l_{2}c_{1}\;p_{k+1}}\\ \;\;\;\;\;B_{\;p_{k+1}}]\\ \;+ig_{1}\left[r_{l_{1}c_{1}\;p_{k};\;l_{2}\;c_{1}\;p_{k}}^{†}\;s_{ep_{k-1}}s_{g\;p_{k-1}}^{†}s_{e\;p_{k}}^{†}r_{l_{1}\;a\;p_{k-1};\;l_{2}b\;p_{k}}\;B_{\;p_{k}}^{†}\right]\\ \;+ig_{2}\left[r_{l_{1}b\;p_{k+2};\;l_{2}\;a\;p_{k+1}}^{†}\;s_{gp_{k+2}}^{†}r_{l_{1}\;c_{1}\;p_{k+1};\;l_{2}c_{1}\;p_{k+1}}\;B_{\;p_{k+1}}\right]\\ \;+ig_{2}\left[r_{l_{1}\;c_{1}p_{k-1};\;l_{2}\;c_{1}\;p_{k-1}}^{†}\;s_{gp_{k-2}}^{†}r_{l_{1}b\;p_{k};\;l_{2}a\;p_{k-1}}\;B_{\;p_{k-1}}^{†}\right]\\ \;+ig_{1}[r_{l_{1}b\;p_{k+1};\;l_{2}\;a\;p_{k}}^{†}\;s_{e\;p_{k}}^{†}s_{ep_{k+1}}\;s_{g\;p_{k+1}}^{†}r_{l_{1}\;c_{2}\;p_{k+1};\;l_{2}c_{2}\;p_{k+1}}\\ \;\;\;\;\;B_{\;p_{k+1}}]\\ \;+ig_{1}\left[r_{l_{1}c_{2}\;p_{k};\;l_{2}\;c_{2}\;p_{k}}^{†}\;s_{ep_{k-1}}s_{g\;p_{k-1}}^{†}s_{e\;p_{k}}^{†}r_{l_{1}b\;p_{k};\;l_{2}a\;p_{k-1}}\;B_{\;p_{k}}^{†}\right]\\ \;+ig_{2}\left[r_{l_{1}\;c_{2}p_{k-1};\;l_{2}\;c_{2}\;p_{k-1}}^{†}\;s_{gp_{k-2}}^{†}r_{l_{1}a\;p_{k-1};\;l_{2}b\;p_{k}}\;B_{\;p_{k-1}}^{†}\right]\\ \;+ig_{2}\left[r_{l_{1}ap_{k+1};\;l_{2}bp_{k+2}}^{†}\;s_{gp_{k+2}}^{†}r_{l_{1}c_{2}\;p_{k+1};\;l_{2}c_{2}\;p_{k+1}}B_{\;p_{k+1}}\;\right]\\ \;-\gamma_{g}\;s_{gp_{k}}^{†}+F_{g}^{†}. \end{array}$$

(4.6) $$\begin{array}{l} {\dot{s}}_{ep_{k}}^{†}=\;ig_{1}\left[r_{l_{1}a\;p_{k-1};\;l_{2}\;b\;p_{k}}^{†}\;s_{gp_{k-1}}s_{e\;p_{k-1}}^{†}s_{gp_{k}}^{†}r_{l_{1}\;c_{1}\;p_{k};\;l_{2}c_{1}\;p_{k}}\;B_{\;p_{k}}\right]\\ \;+ig_{1}[r_{l_{1}c_{1}\;p_{k+1};\;l_{2}\;c_{1}\;p_{k+1}}^{†}\;s_{gp_{k}}^{†}s_{gp_{k+1}}s_{e\;p_{k+1}}^{†}r_{l_{1}a\;p_{k};\;l_{2}b\;p_{k+1}}\\ \;\;\;\;\;\;B_{\;p_{k+1}}^{†}]\\ \;+ig_{1}\left[r_{l_{1}b\;p_{k};\;l_{2}\;a\;p_{k-1}}^{†}s_{gp_{k-1}}\;s_{e\;p_{k-1}}^{†}s_{gp_{k}}^{†}r_{l_{1}\;c_{2}\;p_{k};\;l_{2}c_{2}\;p_{k}}\;B_{\;p_{k}}\right]\\ \;+ig_{1}[r_{l_{1}c_{2}\;p_{k+1};\;l_{2}\;c_{2}\;p_{k+1}}^{†}\;s_{g\;p_{k}}^{†}s_{gp_{k+1}}s_{e\;p_{k+1}}^{†}r_{l_{1}b\;p_{k};\;l_{2}a\;p_{k-1}}\\ \;\;\;\;\;\;B_{\;p_{k+1}}^{†}]-\;\gamma_{e}\;s_{e\;p_{k}}^{†}+F_{e}^{†}. \end{array}$$

(4.7) $$\begin{array}{l} {\dot{B}}_{\;p_{k}}^{†}=\;ig_{1}[\{r_{l_{1}a\;p_{k-1};\;l_{2}\;b\;p_{k}}^{†}r_{l_{1}c_{1}\;p_{k};\;l_{2}\;c_{1}\;p_{k}}\\ \;+r_{l_{1}b\;p_{k};\;l_{2}\;a\;p_{k-1}}^{†}r_{l_{1}c_{2}\;p_{k};\;l_{2}\;c_{2}\;p_{k}}\}\;s_{e\;p_{k-1}}^{†}s_{g\;p_{k-1}}s_{g\;p_{k}}^{†}s_{e\;p_{k}}]\\ \;+ig_{2}[\{r_{l_{1}b\;p_{k+1};\;l_{2}\;a\;p_{k}}^{†}r_{l_{1}c_{1}\;p_{k};\;l_{2}\;c_{1}\;p_{k}}\\ \;+r_{l_{1}a\;p_{k};\;l_{2}\;b\;p_{k+1}}^{†}r_{l_{1}c_{2}\;p_{k};\;l_{2}\;c_{2}\;p_{k}}\}\;s_{g\;p_{k-1}}s_{gp_{k+1}}^{†}]\\ \;-\gamma_{B}\;B_{\;p_{k}}^{†}+F_{B}^{†}. \end{array}$$

To solve the Equations (4.1–4.7), we utilize the semi classical approach and replace the operators by their expectation values in a coherent state representation. In doing so, the operators become complex numbers and the expectation values of the fluctuating forces are zero.

## Solutions

We start the description of the three a fore-mentioned solution with the impact solution and are firstly searching for solutions for each step separately. Later, one overall solution will be outlined by combining all four separate solutions. We are hereby concentrating on one robot walking on one lane. The walking processes of several robots on different lanes will be explained in the succeeding sections.

### Impact solution: first step

As mentioned above, the procedure begins with the first step (Figure 2), at position *k.* The initial state is defined as (starting with position *k* = 1):

(5.1) $$|\phi (t_{0})\rangle =r_{l_{1}a\;p_{1};\;l_{2}\;bp_{2}}^{†}s_{e\;p_{1}}^{†}s_{g\;p_{2}}^{†}|\phi_{0}\rangle ,$$

where | ϕ_0_ 〉 delineates the vacuum state. The initial conditions of all relevant states are:

(5.2) $$r_{l_{1}a\;p_{1};\;l_{2}bp_{2}}^{†}\left(t_{0}\right)=1,r_{l_{1}c_{1}p_{2};\;l_{2}c_{1}p_{2}}^{†}(t_{0})=0.$$

(5.3) $$\begin{array}{l} s_{ep_{1}}^{†}\left(t_{0}\right)=1,s_{gp_{1}}^{†}\left(t_{0}\right)=0,s_{gp_{2}}^{†}\left(t_{0}\right)=1,s_{ep_{2}}^{†}\left(t_{0}\right)=0.\;\\ B_{p_{2}}^{†}\left(t_{0}\right)=0. \end{array}$$

We set $r_{l_{1}c_{2}p_{1};\;l_{2}c_{2}\;p_{1}}^{†}$ = 0 in Equation (4.1) because this state is not present. The resulting modified Equation (5.1) now reads:

(5.4) $$\begin{array}{l} {\dot{r}}_{l_{1}a\;p_{1};\;l_{2}\;b\;p_{2}}^{†}=\;ig_{1}\left[s_{ep_{1}}s_{gp_{1}}^{†}r_{l_{1}c_{1}p_{2};\;l_{2}c_{1}p_{2}}^{†}s_{gp_{2}}s_{ep_{2}}^{†}B_{p_{2}}^{†}\right]\\ \;-\;\gamma_{ab}r_{l_{1}a\;p_{1};\;l_{2}b\;p_{2}}^{†}. \end{array}$$

In a similar way, going through all remaining equations the following reduced set of equations is obtained:

(5.5) $$\begin{array}{l} {\dot{r}}_{l_{1}c_{1}p_{2};\;l_{2}c_{1}p_{2}}^{†}=\;ig_{1}\left[s_{ep_{1}}^{†}s_{gp_{1}}r_{l_{1}a\;p_{1};\;l_{2}bp_{2}}^{†}s_{gp_{2}}^{†}s_{ep_{2}}B_{p_{2}}\right]\\ \;-\;\gamma_{c_{1}}r_{l_{1}c_{1}\;p_{2};\;l_{2}c_{1}p_{2}}^{†} \end{array}$$

(5.6) $${\dot{s}}_{gp_{1}}^{†}=\;ig_{1}\left[r_{l_{1}ap_{1};l_{2}b\;p_{2}}^{†}s_{ep_{1}}^{†}s_{gp_{2}}^{†}s_{e\;p_{2}}r_{l_{1}c_{1}p_{2};l_{2}c_{1}p_{2}}B_{p_{2}}\right]-\gamma_{g}s_{gp_{1}}^{†},$$

(5.7) $${\dot{s}}_{gp_{2}}^{†}=\;ig_{1}\left[r_{l_{1}c_{1}\;p_{2};\;l_{2}\;c_{1}\;p_{2}}^{†}s_{ep_{1}}s_{g\;p_{1}}^{†}s_{e\;p_{2}}^{†}r_{l_{1}\;a\;p_{1};\;l_{2}b\;p_{2}}B_{p_{2}}^{†}\right]-\gamma_{g}s_{gp_{2}}^{†},$$

(5.8) $${\dot{s}}_{ep_{1}}^{†}=\;ig_{1}\left[\;r_{l_{1}c_{1}p_{2};l_{2}c_{1}p_{2}}^{†}s_{gp_{1}}^{†}s_{gp_{2}}\;s_{ep_{2}}^{†}r_{l_{1}ap_{1};l_{2}bp_{2}}B_{p_{2}}^{†}\right]-\gamma_{e}s_{ep_{1}}^{†}$$

(5.9) $${\dot{s}}_{ep_{2}}^{†}=\;ig_{1}\left[r_{l_{1}a\;p_{1};\;l_{2}b\;p_{2}}^{†}\;s_{gp_{1}}s_{ep_{1}}^{†}s_{gp_{2}}^{†}r_{l_{1}c_{1}\;p_{2};\;l_{2}c_{1}\;p_{2}}\;B_{p_{2}}\right]-\gamma_{e}s_{ep_{2}}^{†}.$$

(5.10) $${\dot{B}}_{p_{2}}^{†}=\;ig_{1}\left[r_{l_{1}ap_{1};\;l_{2}\;bp_{2}}^{†}\;s_{g\;p_{1}}s_{gp_{2}}^{†}s_{e\;p_{1}}^{†}s_{e\;p_{2}}r_{l_{1}c_{1}\;p_{2};\;l_{2}\;c_{1}p_{2}}\right]-\gamma_{B}B_{p_{2}}^{†}.$$

Before presenting the complete set of numerical solutions for all Equations (5.4–5.10), we prefer to assure ourselves that the right solution can also be generated by analytical methods and not only by numerical calculations. This should convince us that we are on the right track.

The following approach (setting equally the robot-relevant damping constants γ = γ_*ab*_ = γ_*ba*_ = γ_*c*_1__ = γ_*c*_2__, and neglecting for the moment the damping of the *B* operator: γ_*B*_ = 0), delivers a consistent, periodic solution for a walking biped molecular robot:

(5.11) $$r_{l_{1}ap_{1};l_{2}bp_{2}}^{†}=\cos(f)e^{-\gamma t},r_{l_{1}c_{1}p_{2};l_{2}c_{1}p_{2}}^{†}=\sin(f)e^{-\gamma t}.$$

(5.12) $$s_{ep_{1}}^{†}=\cos(g),s_{gp_{1}}^{†}=i\sin(g),s_{ep_{2}}^{†}=\cos(h),s_{gp_{2}}^{†}=\sin(h).$$

(5.13) $$B_{p_{2}}^{†}=b,\;b\;\in \mathbb{R}.$$

By inserting these expressions into the Equations (5.4–5.10), four coupled equations for the four variables *f*, *g*, *h*, *b* are finally obtained:

(5.14) $$\dot{f}=\;\frac{g_{1}}{4}\sin\left(2g\right)\sin\left(2h\right)b.$$

(5.15) $$\dot{g}=\;\frac{g_{1}}{4}\sin\left(2f\right)e^{-\gamma t}\sin\left(2h\right)b.$$

(5.16) $$\dot{h}=\;-\frac{g_{1}}{4}\sin(2f)e^{-\gamma t}\sin\left(2g\right)b.$$

(5.17) $$\dot{b}=\;\frac{g_{1}}{2}\dot{g}\;\sin\left(2g\right).$$

The last equation can be directly solved by the technique of separation of variables:

(5.18) $$b\left(t\right)=\;-\frac{g_{1}}{4}\left[\cos\left(2g\left(t\right)\right)-\cos\left(2g\left(t_{0}\right)\right)\right]+b\left(t_{0}\right).$$

The calculation is started with the assumption that γ = 0 is valid in order to start with “perfect” symmetry. Afterwards, we will switch to the damping process in order to observe how this symmetry will be broken.

**Figure 3:**
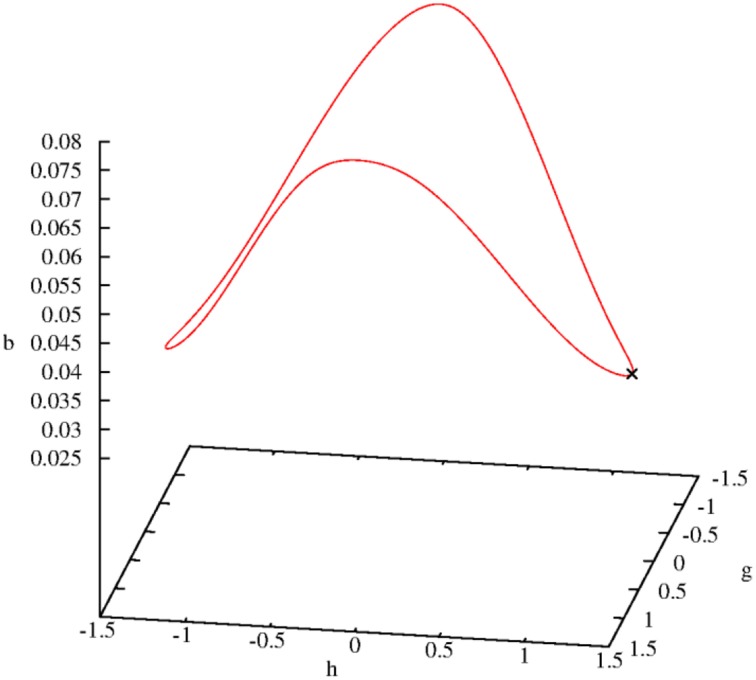
**Three-dimensional limit cycle defined by the three variables *b*, *g*, and *h***. The initial point is marked by a cross.

**Figure 4:**
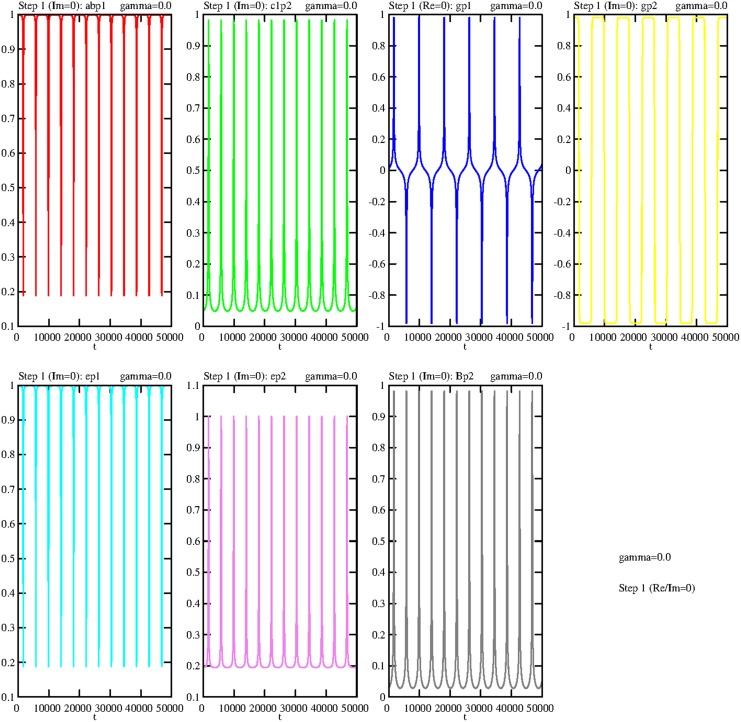
**Temporal dependence of the real parts of all variables of step 1, with γ = 0**. The algebraic symbols are: $\;abp1=r_{l_{1}ap_{1};l_{2}bp_{2}}^{†},\;c1p2=r_{l_{1}c_{1}p_{2};l_{2}c_{1}p_{2}}^{†},\;gp1=s_{gp_{1}}^{†},\;gp2=s_{gp_{2}}^{†},\;ep1=s_{ep_{1}}^{†},\;ep2=s_{ep_{2}}^{†},\;Bp2=B_{p_{2}}^{†}$. The coupling constants are *g*_1_ = *g*_2_ = 0.1.

Incidentally, it is not surprising if all solutions of the Equations (5.14–5.17) are periodic (wave-like) if the damping process is switched off. Figure 3 demonstrates this prediction in a phase portrait (Holmes et al., 2012) that also includes the expected periodicity of the variable b (*B*-field):

Even if the general complex solutions of the Equations (5.4–5.10) are calculated, symmetrical patterns are visible. Figure 4 shows the real parts of all operators with the expected symmetry. The imaginary parts show a very similar behavior; therefore they are not presented here or in the next sections.

It is well known that processes without any damping are artifacts. Therefore, we will now include damping processes. Even if “perfect symmetry” is not realistic, it is worth taking it into consideration since it serves as a kind of “roadmap” to the new individual trajectories if γ deviates from zero and becomes positive.

Even if the damping constant is assigned the small value γ = 0.0005 the symmetry (periodicity) will be broken. The limit cycle shown in Figure 3 is dissolved and the trajectory goes directly to zero (fixed point). The same happens with the general solution—it goes directly to zero. Thus, in both cases a large γ value should be chosen.

If the value of γ is continuously decreased, the ringing effect increases along the trajectory to γ = 0. Therefore, we can achieve all possible trajectory patterns, from a direct path to zero to complete oscillation (no damping).

**Figure 5:**
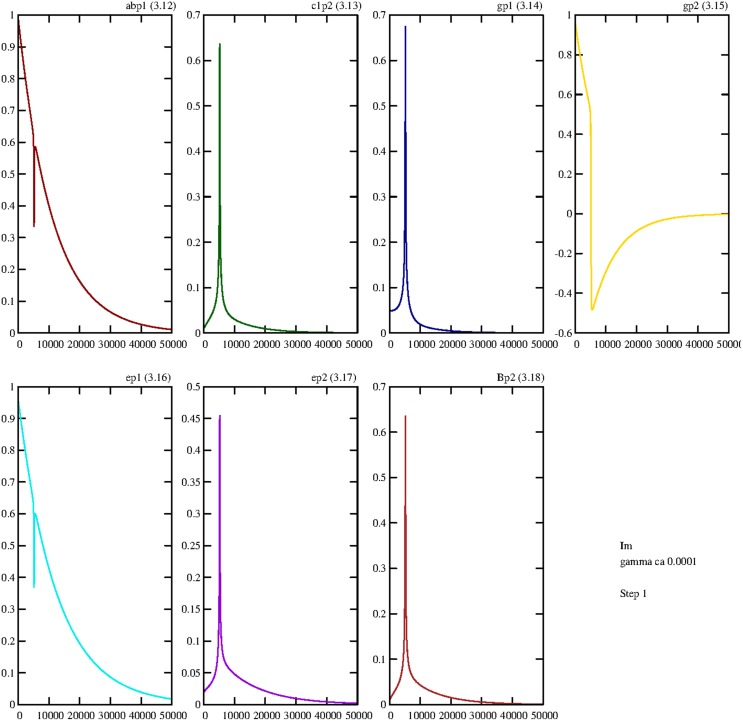
**Damped temporal curves representing the real parts of the following operators of the first step: $abp1=r_{l_{1}ap_{1};l_{2}bp_{2}}^{†},c1p2=r_{l_{1}c_{1}p_{2};l_{2}c_{1}p_{2}}^{†},gp1=s_{gp_{1}}^{†},gp2=s_{gp_{2}}^{†},ep1=s_{ep_{1}}^{†},ep2=s_{ep_{2}}^{†},Bp2=B_{p_{2}}^{†}$**. The value of the damping constant is set to γ = 0.0001. The coupling constants are *g*_1_ = *g*_2_ = 0.1.

Even if the value of the damping constant slightly diminishes, to γ = 0.0001, the first effect can be observed; after the first peak the trajectory converges to zero (Figure 5).

The results of all remaining steps, 2–4, will not be presented in the same detail as in step 1. Only the actual initial conditions and the corresponding equations of motion are given. At the end of this subchapter, we present all four steps in sequence.

### Impact solution: second step

The description continues with a presentation of the second step. For reasons of clarity, we set *k* + 1 = 2; the initial state is defined by:

(5.19) $$|\phi (t_{1}))\rangle =r_{l_{1}c_{1}p_{2};l_{2}c_{1}p_{2}}^{†}s_{e\;p_{2}}^{†}B_{p_{2}}^{†}|\phi_{0}\rangle ,$$

The initial conditions are:

(5.20) $$r_{l_{1}c_{1}p_{2};\;l_{2}c_{1}\;p_{2}}^{†}(t_{1})=1,r_{l_{1}b\;p_{3};\;l_{2}a\;p_{2}}^{†}\left(t_{1}\right)=0.$$

(5.21) $$\begin{array}{l} s_{gp_{1}}^{†}\left(t_{1}\right)=1,s_{ep_{2}}^{†}\left(t_{1}\right)=1,s_{gp_{3}}^{†}\left(t_{1}\right)=0.\\ B_{p_{2}}^{†}\left(t_{1}\right)=1. \end{array}$$

The Heisenberg equations of motion of the expectation values of step 2 are:

(5.22) $${\dot{r}}_{l_{1}c_{1}\;p_{2};\;l_{2}\;c_{1}\;p_{2}}^{†}=\;ig_{2}\left[s_{gp_{1}}s_{gp_{3}}^{†}r_{l_{1}b\;p_{3};\;l_{2}a\;p_{2}}^{†}B_{p_{2}}\right]-\gamma_{c_{1}}r_{l_{1}c_{1}\;p_{2};\;l_{2}\;c_{1}\;p_{2}}^{†}.$$

(5.23) $${\dot{r}}_{l_{1}b\;p_{3};\;l_{2}\;ap_{2}}^{†}=\;ig_{2}\left[s_{gp_{1}}^{†}s_{g\;p_{3}}r_{l_{1}c_{1}\;p_{2};\;l_{2}\;c_{1}p_{2}}^{†}\;B_{p_{2}}^{†}\right]-\gamma_{ba}r_{l_{1}b\;p_{3};\;l_{2}ap_{2}}^{†}$$

(5.24) $${\dot{s}}_{gp_{1}}^{†}=\;ig_{2}\left[r_{l_{1}bp_{3};\;l_{2}ap_{2}}^{†}s_{gp_{3}}^{†}r_{l_{1}\;c_{1}p_{2};\;l_{2}c_{1}p_{2}}B_{p_{2}}\right]-\gamma_{g}s_{gp_{1}}^{†}.$$

(5.25) $${\dot{s}}_{gp_{3}}^{†}=\;ig_{2}\left[r_{l_{1}\;c_{1}p_{2};\;l_{2}\;c_{1}\;p_{2}}^{†}\;s_{gp_{1}}^{†}r_{l_{1}b\;p_{3};\;l_{2}a\;p_{2}}B_{p_{2}}^{†}\right]-\gamma_{g}s_{gp_{3}}^{†}.$$

(5.26) $${\dot{B}}_{p_{2}}^{†}=\;ig_{2}[r_{l_{1}b\;p_{3};\;l_{2}\;ap_{2}}^{†}s_{gp_{1}}s_{gp_{3}}^{†}r_{l_{1}c_{1}\;p_{2};\;l_{2}\;c_{1}p_{2}}]-\gamma_{B}B_{p_{2}}^{†}.$$

**Figure 6:**
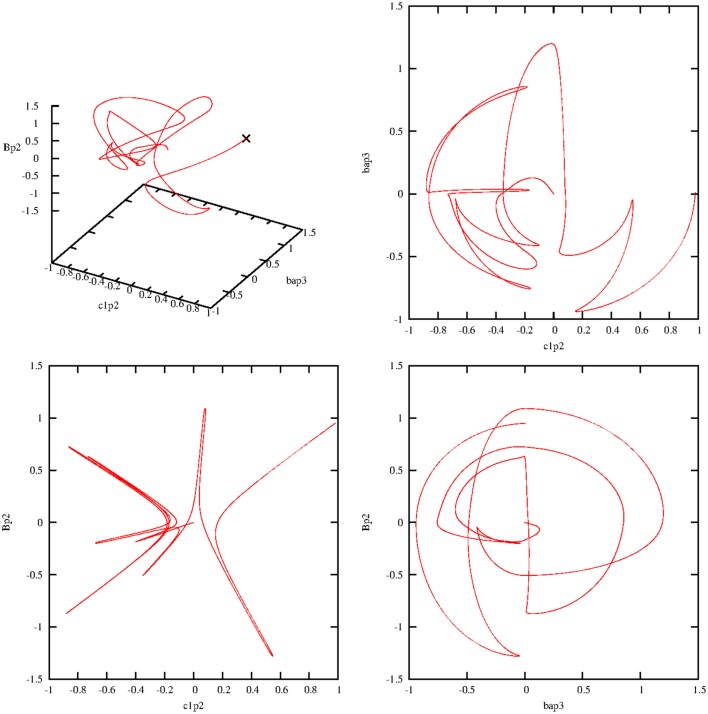
**Phase portraits of the real parts of the second step of the following operators: $bap3=r_{l_{1}ap_{3};l_{2}bp_{2}}^{†},\;c1p2=r_{l_{1}c_{1}p_{2};l_{2}c_{1}p_{2}}^{†},\;gp1=s_{gp_{1}}^{†},\;gp3=s_{gp_{3}}^{†},\;Bp2=B_{p_{2}}^{†}$**. The value of the damping constant is γ = 0.0005. All trajectories vary after their starting points (marked by a cross in the first figure) until they end up at the fixed point 0. The coupling constants are *g*_1_ = *g*_2_ = 0.1.

Here, the damping process is turned on again at the beginning, with γ = 0.0005. Figure 6 shows the expected “ringing” effects now represented in phase portraits.

### Impact solution: third step

The presentation continues with a description of the third step. For reasons of comprehensibility better readability, we set *k* + 2 = 3. The initial state is fixed by:

(5.27) $$|\phi (t_{2}))\rangle =r_{l_{1}b\;p_{3};\;l_{2}a\;p_{2}}^{†}s_{e\;p_{2}}^{†}s_{g\;p_{3}}^{†}|\phi_{0}\rangle ,$$

In this case, the initial conditions of all states are:

(5.28) $$r_{l_{1}b\;p_{3};\;l_{2}\;a\;p_{2}}^{†}\left(t_{2}\right)=1,r_{l_{1}c_{2}p_{3};\;l_{2}c_{2}\;p_{3}}^{†}(t_{2})=0,$$

(5.29) $$s_{ep_{2}}^{†}\left(t_{2}\right)=1,s_{gp_{2}}^{†}\left(t_{2}\right)=0,s_{ep_{3}}^{†}\left(t_{2}\right)=0,s_{gp_{3}}^{†}\left(t_{2}\right)=1.$$

(5.30) $$B_{p_{3}}^{†}\left(t_{2}\right)=0.$$

The Heisenberg equations of motion of the expectation values of step 3 are:

(5.31) $$\begin{array}{l} {\dot{r}}_{l_{1}b\;p_{3};\;l_{2}ap_{2}}^{†}=\;ig_{1}\left[s_{e\;p_{2}}s_{gp_{2}}^{†}r_{l_{1}c_{2}\;p_{3};\;l_{2}\;c_{2}\;p_{3}}^{†}s_{gp_{3}}s_{ep_{3}}^{†}\;B_{p_{3}}^{†}\right]\\ \;-\gamma_{ba}r_{l_{1}b\;p_{3};\;l_{2}ap_{2}}^{†} \end{array}$$

(5.32) $$\begin{array}{l} {\dot{r}}_{l_{1}c_{2}\;p_{3};\;l_{2}c_{2}\;p_{3}}^{†}=\;ig_{1}\left[s_{gp_{2}}\;s_{ep_{2}}^{†}r_{l_{1}b\;p_{3};\;l_{2}\;a\;p_{2}}^{†}s_{ep_{3}}s_{gp_{3}}^{†}B_{p_{3}}\right]\\ \;-\gamma_{c_{2}}r_{l_{1}c_{2}\;p_{3};\;l_{2}\;c_{2}\;p_{3}}^{†} \end{array}$$

(5.33) $${\dot{s}}_{gp_{2}}^{†}=\;ig_{1}\left[r_{l_{1}bp_{3};l_{2}\;ap_{2}}^{†}s_{ep_{2}}^{†}s_{ep_{3}}\;s_{gp_{3}}^{†}r_{l_{1}\;c_{2}\;p_{3};l_{2}c_{2}\;p_{3}}\;B_{p_{3}}\right]-\gamma_{g}\;s_{gp_{2}}^{†}.$$

(5.34) $${\dot{s}}_{gp_{3}}^{†}=\;ig_{1}\left[r_{l_{1}c_{2}\;p_{3};\;l_{2}\;c_{2}\;p_{3}}^{†}s_{ep_{2}}s_{gp_{2}}^{†}s_{ep_{3}}^{†}r_{l_{1}b\;p_{3};\;l_{2}a\;p_{2}}\;B_{p_{3}}^{†}\right]\;-\;\gamma_{g}\;s_{gp_{3}}^{†}.$$

(5.35) $${\dot{s}}_{ep_{2}}^{†}=\;ig_{1}\left[r_{l_{1}c_{2}p_{3};\;l_{2}c_{2}p_{3}}^{†}\;s_{gp_{2}}^{†}s_{gp_{3}}s_{ep_{3}}^{†}r_{l_{1}b\;p_{3};\;l_{2}a\;p_{2}}B_{p_{3}}^{†}\right]-\;\gamma_{e}\;s_{ep_{2}}^{†}.$$

(5.36) $${\dot{s}}_{ep_{3}}^{†}=\;ig_{1}\left[r_{l_{1}b\;p_{3};\;l_{2}\;a\;p_{2}}^{†}s_{gp_{2}}\;s_{ep_{2}}^{†}s_{gp_{3}}^{†}r_{l_{1}\;c_{2}\;p_{3};\;l_{2}c_{2}\;p_{3}}\;B_{p_{3}}\right]-\;\gamma_{e}\;s_{ep_{3}}^{†}.$$

(5.37) $${\dot{B}}_{p_{3}}^{†}=\;ig_{1}\left[\;r_{l_{1}b\;p_{3};\;l_{2}\;a\;p_{2}}^{†}r_{l_{1}c_{2}\;p_{3};\;l_{2}\;c_{2}\;p_{3}}\;s_{ep_{2}}^{†}\;s_{gp_{2}}\;s_{g\;p_{3}}^{†}s_{e\;p_{3}}\right]-\;\gamma_{B}B_{p_{3}}^{†}.$$

Attention is drawn to the symmetry that exists between all equations of motion of the first and third steps. Further, the initial condition for *s*^†^_*gp*_2__ in step 3 is *s*^†^_*gp*_2__(*t*_2_) = 0, while in step 1 *s*^†^_*gp*_2__(*t*_0_) = 1 was valid. Similarly, the value *s*^†^_*ep*_2__(*t*_2_) = 1 is assumed in step 3, whereas *s*^†^_*ep*_2__(*t*_0_) = 0 was correct in step 1.

### Impact solution: fourth step

The presentation continues with a description of the fourth step. Here, we set +2 = 4, and the initial state is given by:

(5.38) $$|\phi (t_{3}))\rangle =r_{l_{1}c_{2}p_{3};\;l_{2}c_{2}p_{3}}^{†}s_{ep_{3}}^{†}B_{p_{3}}^{†}|\phi_{0}\rangle ,$$

where | ϕ_0_ 〉 defines the vacuum state. In this case, the initial conditions of the two robot states are:

(5.39) $$r_{l_{1}b\;p_{3};\;l_{2}\;a\;p_{2}}^{†}\left(t_{3}\right)=0,r_{l_{1}c_{2}p_{3};\;l_{2}c_{2}\;p_{3}}^{†}(t_{3})=1,.$$

The initial states of the substrate at the three positions *p*_2_, *p*_3_ and *p*_4_ are:

(5.40) $$s_{gp_{2}}^{†}\left(t_{3}\right)=1,s_{ep_{3}}^{†}\left(t_{3}\right)=1,s_{gp_{4}}^{†}\left(t_{3}\right)=0.$$

The heat-bath operator satisfies the initial condition *B*^†^_*p*__3_(*t*_3_) = 1.

The relevant Heisenberg equations of motion of step 4 are:

(5.41) $${\dot{r}}_{l_{1}c_{2}\;p_{3};\;l_{2}\;c_{2}p_{3}}^{†}=\;ig_{2}\left[s_{gp_{2}}^{†}s_{gp_{4}}r_{l_{1}a\;p_{3};\;l_{2}bp_{4}}^{†}B_{p_{3}}\right]-\;\gamma_{c_{2}}r_{l_{1}c_{2}\;p_{3};\;l_{2}\;c_{2}\;p_{3}}^{†}.$$

(5.42) $${\dot{r}}_{l_{1}a\;p_{3};\;l_{2}b\;p_{4}}^{†}=\;ig_{2}\left[s_{gp_{2}}^{†}s_{gp_{4}}r_{l_{1}c_{2}\;p_{3};\;l_{2}\;c_{2}\;p_{3}}^{†}B_{p_{3}}^{†}\right]-\;\gamma_{ab}r_{l_{1}ap_{3};\;l_{2}bp_{4}}^{†}.$$

(5.43) $${\dot{s}}_{gp_{2}}^{†}=\;ig_{2}\left[r_{l_{1}a\;p_{3};\;l_{2}\;b\;p_{4}}^{†}\;s_{gp_{4}}^{†}r_{l_{1}c_{2}\;p_{3};\;l_{2}c_{2}\;p_{3}}B_{p_{3}}\right]\;-\;\gamma_{g}s_{gp_{2}}^{†},$$

(5.44) $${\dot{s}}_{gp_{4}}^{†}=\;ig_{2}\left[r_{l_{1}c_{2}\;p_{3};\;l_{2}\;c_{2}\;p_{3}}^{†}s_{gp_{2}}^{†}r_{l_{1}ap_{3};\;l_{2}b\;p_{4}}B_{p_{3}}^{†}\right]-\;\gamma_{g}s_{gp_{4}}^{†}$$

(5.45) $${\dot{B}}_{p_{3}}^{†}=\;ig_{2}\left[r_{l_{1}a\;p_{3};\;l_{2}\;b\;p_{4}}^{†}r_{l_{1}c_{2}\;p_{3};\;l_{2}\;c_{2}\;p_{3}}s_{gp_{2}}s_{gp_{4}}^{†}\right]-\;\gamma_{B}B_{p_{3}}^{†}.$$

### Impact solution: all four steps in succession

**Figure 7:**
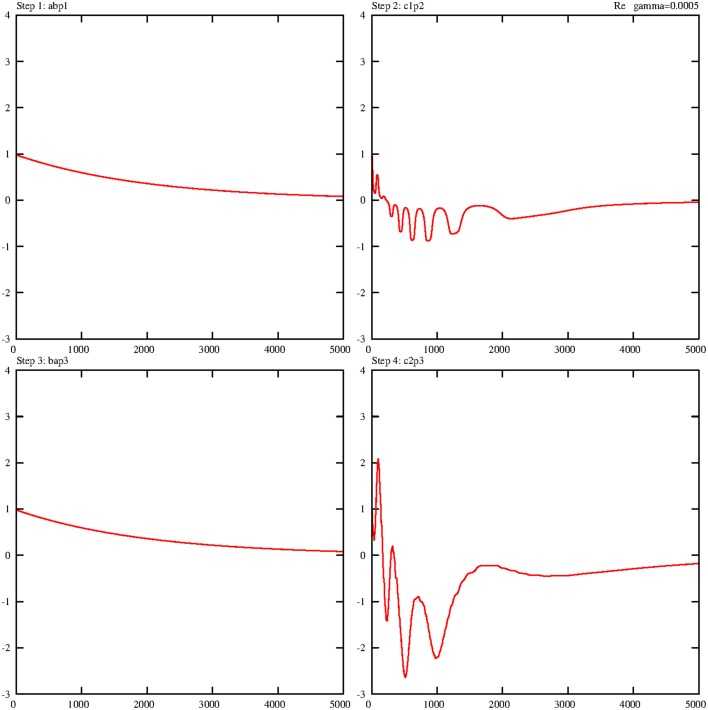
**Damped temporal curves representing the real parts of the four molecular robot operators that are consecutively performed by the steps 1–4**. The following algebraic equivalents for the selected operators are introduced: $abp1=r_{l_{1}ap_{1};l_{2}bp_{2}}^{†},\;c1p2=r_{l_{1}c_{1}p_{2};l_{2}c_{1}p_{2}}^{†},\;bap3=r_{l_{1}bp_{3};l_{2}ap_{2}}^{†},\;c2p3=r_{l_{1}c_{2}p_{3};l_{2}c_{2}p_{3}}^{†}$. The value of the damping constant is set to γ = 0.0005. The coupling constants are *g*_1_ = *g*_2_ = 0.1.

Now all four partial solutions are collected and put together into one sequence. The first consecutive view describes the changes in the four robot states during the four separate walking steps. Figure 7 illustrates the corresponding real parts (imaginary parts are again very similar, therefore their representation is skipped) of the transitions of these walking states on a reduced scale of 5000 steps for better visibility. Clearly, two typical effects are observable. The similar steps 1 and 3 are regularly performed and go directly from their initial values to zero. Steps 2 and 4 also conform to this pattern, but one on a broader scale, because step 4 shows for $r_{l_{1}c_{1}p_{2};l_{2}c_{1}p_{2}}^{†}$ slightly more oscillations than for $r_{l_{1}ap_{1};l_{2}bp_{2}}^{†}$ in step 2.

**Figure 8:**
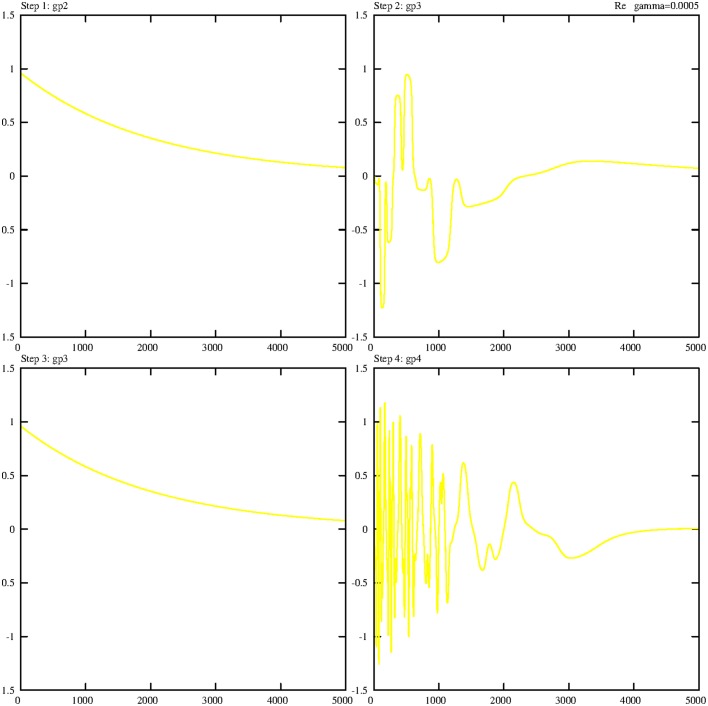
**Damped temporal curves representing the real parts of three selected substrate operators (ground states) that are consecutively performed by steps 1–4**. The following algebraic equivalents for the selected operators are introduced: *gp*2 = *s*^†^_*gp*_2__, *gp*3 = *s*^†^_*gp*_3__, *gp*4 = *s*^†^_*gp*_4__. The value of the damping constant is set to γ = 0.0005. The coupling constants are *g*_1_ = *g*_2_ = 0.1.

Figure 8 summarizes the sequence of the real parts of the ground states of the substrate for all four steps. Here another effect comes to light. The behavior of *s*^†^_*gp*__3_ is different in step 2, and during step 2 (see Figure 2), the ground state *s*^†^_*gp*__3_ does not exist and has to be generated, whereas during step 3 this ground state already exists and must be annihilated and transformed into an exited state.

From a purely mathematical point of view, we have to compare the two Equations (5.25) and (5.34) for *s*^†^_*gp*__3_ in the second and third steps. In the second step, there is a strong mutual dependence between *s*^†^_*gp*__3_ and *s*^†^_*gp*__1_. In Equation (5.34), we mainly have to consider the dependence of *s*^†^_*gp*__3_ and *s*^†^_*ep*__3_. The operator product *s*_*ep*_2__*s*^†^_*gp*_2__ is time-independent and self-consistent, while substrate states g and e produced themselves reciprocally (see also Figure 10). The three remaining bordering operators (2 *r*-operators, one *B*-operator) are equivalent in both expressions.

**Figure 9:**
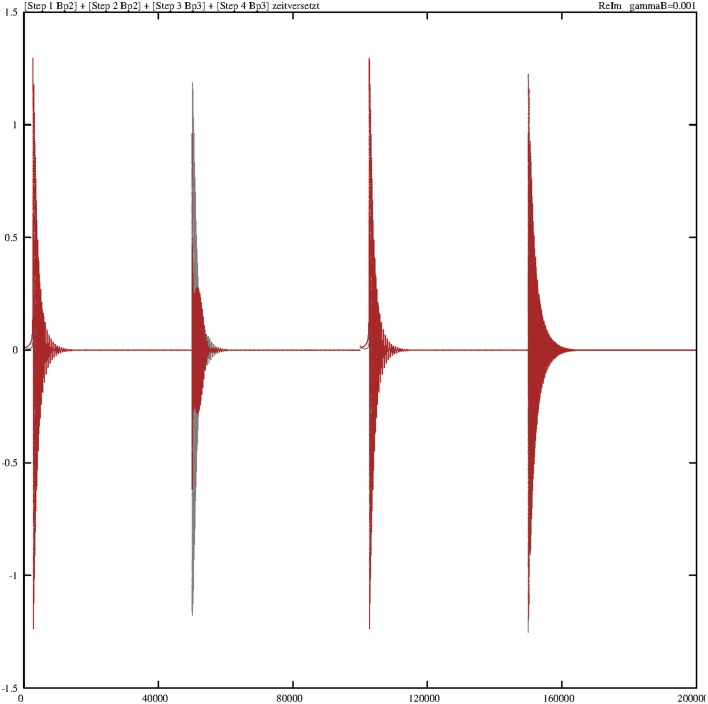
**Damped temporal curves showing the overlay of the real (gray) and imaginary (red) parts of the operators *B*^†^_*p*_2__ and *B*^†^_*p*_3__, which are consecutively outlined through steps 1–4**. The algebraic equivalents of the selected operators are: *Bp*2 = *B*^†^_*p*_2__, *Bp*3 = *B*^†^_*p*_3__. The damping constant is set to γ_*B*_ = 0.001. The coupling constants are *g*_1_ = *g*_2_ = 0.1.

The next focus of attention is the behavior of the synchronizing heat-bath operators *B*^†^_*p*__2_ and *B*^†^_*p*__3_. Here, we consider at first the effect of the damping value γ_*B*_ = 0.001 and fix all other damping constants to zero. Figure 9 reveals the behavior of the overlay of the real parts (gray) and imaginary parts (red) of these two operators. Concerning the real parts, both operators are “strong” during steps 2 and 4 (the fueling process) and “weak” in the two other steps. During step 1, *B*^†^_*p*__2_ starts with *B*^†^_*p*__2_(*t*_0_) = 0 and ends with *B*^†^_*p*__2_(*t*_1_) = 1. In step 2, it starts with this initial value, *B*^†^_*p*__2_(*t*_2_) = 1, and ends permanently with *B*^†^_*p*__2_(*t*_2_) = 0. Such interplay is valid in the same manner for *B*^†^_*p*__3_(*t*_3_). The trajectories of the imaginary parts of these two operators are distinct in that way that the real parts of these two operators in steps 2 and 4 are no longer dominant.

**Figure 10:**
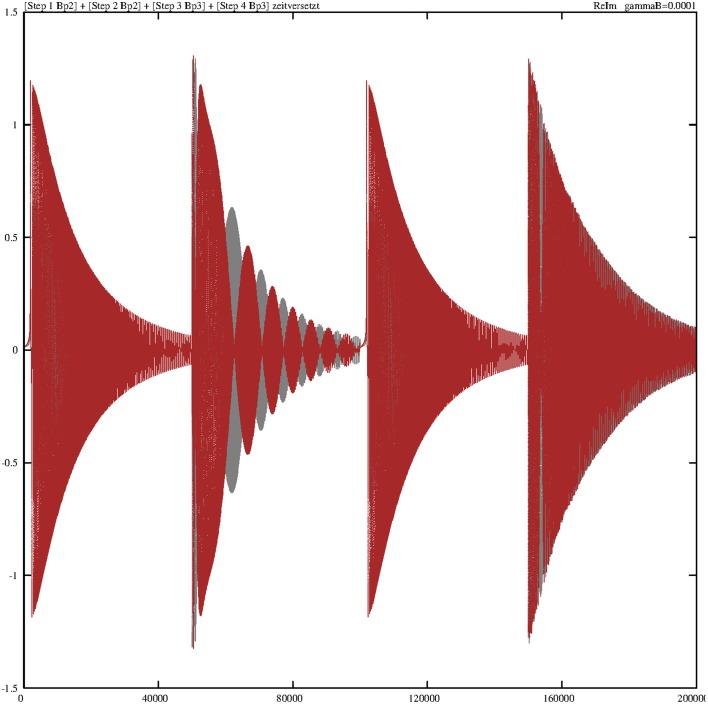
**Damped temporal curves showing the overlay of the real (gray) and imaginary (red) parts of the operators *B*^†^_*p*_2__ and *B*^†^_*p*_3__, which are consecutively outlined through steps 1–4**. The algebraic equivalents of the selected operators are *Bp*2 = *B*^†^_*p*_2__ and *Bp*3 = *B*^†^_*p*_3__. The value of the damping constant is γ_*B*_ = 0.0001. The coupling constants are *g*_1_ = *g*_2_ = 0.1.

The situation greatly changes if we decrease the corresponding damping constant. Figure 10 portrays the temporal diagram of the overlay of the real and imaginary parts of *B*^†^_*p*__2_ and *B*^†^_*p*__3_. The contours of each step can be considered as an envelope around the oscillating solutions.

The next two sections focus on wave solutions. Firstly, we combine modes that synchronize within a lane and between lanes (similar to light modes or laser modes, e.g., Haken, 1970). Secondly, we present a solution that constitutes a running wave.

### Synchronized wave-based motion

#### Coherent motion on parallel substrate lanes

It is assumed that there are *M* different lanes, and on each lane up to *L* positions (lane length) are available. The different walking lanes of robots are distinguished by the index *m* = 1, …, *M*, and the position of a molecular robot on lane *m* is denoted by *p*_*m*_ = 1, …, *L*. The *B* fields are also coupled in a lane and across different lanes. Therefore, we introduce a new two-dimensional vector *l* by combining both parameters *l* = (*m*, *p*_*m*_). This combination modifies the Equations (4.1–4.7) by an additional index, as e.g., demonstrated by the reformulation of Equation (4.1):

(5.46) $$\begin{array}{l} {\dot{r}}_{l_{1}al;\;l_{2}bl+(0,1)}^{†}=\;ig_{1}[s_{el}\;s_{gl}^{†}r_{l_{1}c_{1}l+(0,1);\;l_{2}\;c_{1}l+(0,1)}^{†}s_{gl+(0,1)}\;s_{el+(0,1)}^{†}\\ \;\;B_{l+(0,1)}^{†}]+ig_{2}\left[s_{gl-(0,1)}^{†}\;s_{gl+(0,1)}\;r_{l_{1}c_{2}\;l;\;l_{2}\;c_{2}l}^{†}B_{l}^{†}\right]\\ \;-\gamma_{ab,l}\;r_{l_{1}al;\;l_{2}bl+(0,1)}^{†}+\;F_{ab,\;l}^{†} \end{array}$$

Further, we define another two-dimensional vector $k=\left(k_{j}=\frac{2\pi \;n_{j}}{L},\;k_{j+1}=\frac{2\pi \;n_{j+1}}{L}\right),\;n_{j},\;n_{j+1}=\ldots ,-1,\;0,\;1,\;\ldots$ which defines a two-dimensional wave number vector. This second vector has been introduced since the operator *B*^†^_*l*_ will be developed by a plane wave approximation:

(5.47) $$B_{l}^{†}=\frac{1}{\sqrt{L}}\;\sum_{k}{\tilde{B}}_{k}^{†}e^{ik\cdot l}.$$

The synchronization activities of the two heat-bath operators *B*^†^_*l*_, *B*_*l*_ on the molecular robots are described as external signal field modes that are coupled by the constant *J*_*ll*′_.

We insert this hypothesis in the “parallel” extension of Equation (4.7), indicating that our approach now applies to parallel *M* lanes. This therefore implies that *l* is fixed and over all paths we sum up *m* at the various positions with respect to the fixed position *p*_*m*_ of *l*. The use of the coupling constant *J*_*ll*′_ for $\;{\tilde{B}}_{k}^{†}$ requests a summation along a lane and between lanes:

(5.48) $$\begin{array}{l} B_{l}^{†}=\;\frac{1}{\sqrt{L}}\sum_{k}{\dot{\tilde{B}}}_{k}^{†}e^{ik\cdot l}\\ \;=\;ig_{1}[\sum_{m}\{r_{(l_{1}ap_{m-1};\;l_{2}bp_{m}),\;m}^{†}\;r_{(l_{1}c_{1}p_{m};\;l_{2}\;c_{1}p_{m}),m}\\ \;+\;r_{(l_{1}bp_{m};\;l_{2}\;ap_{m-1}),\;m}^{†}\;r_{(l_{1}c_{2}\;p_{m};\;l_{2}\;c_{2}p_{m}),m}\}\\ \;\times \;s_{ep_{m-1},m}^{†}s_{gp_{m-1},m}\;s_{gp_{m},m}^{†}s_{ep_{m},m}]\\ \;+\;ig_{2}[\sum_{m}\{r_{(l_{1}b\;p_{m+1};\;l_{2}\;ap_{m}),m}^{†}\;r_{(l_{1}c_{1}\;p_{m};\;l_{2}\;c_{1}p_{m}),m}\\ \;+\;r_{(l_{1}a\;p_{m};\;l_{2}bp_{m+1}),m}^{†}\;r_{(l_{1}c_{2}\;p_{m};\;l_{2}c_{2}p_{m}),m}\}\;s_{gp_{m-1}}s_{gp_{m+1},m}^{†}]\\ \;-\;\gamma_{B,\;l}\frac{1}{\sqrt{L}}\;\sum_{k}{\tilde{B}}_{k}^{†}e^{ik\cdot l}+F_{B,l}^{†}+\frac{1}{\sqrt{L}}\sum_{l^{\prime }}J_{ll^{\prime }}\;\sum_{k}{\tilde{B}}_{k}^{†}e^{ik\cdot l^{\prime }}. \end{array}$$

We multiply both sides of (5.48) with $\frac{1}{\sqrt{L}}\sum_{l}e^{-ik^{\prime }\cdot l}$ and use the orthogonal relation $\frac{1}{L}\sum_{l}e^{i(k\cdot l-k^{\prime }\cdot l)}=\delta_{kk^{\prime }}$:

(5.49) $$\begin{array}{l} {\dot{\tilde{B}}}_{k^{\prime }}^{†}=\;ig_{1}[\sum_{l,m}\frac{1}{\sqrt{L}}e^{-ik^{\prime }\cdot l}\{r_{\left(l_{1}ap_{m-1};\;l_{2}bp_{m}\right),m}^{†}r_{(l_{1}c_{1}p_{m};l_{2}c_{1}p_{m}),m}\\ \;+\;r_{\left(l_{1}bp_{m};\;l_{2}\;ap_{m-1}\right),\;m}^{†}\;r_{(l_{1}c_{2}\;p_{m};\;l_{2}\;c_{2}p_{m}),m}\}\\ \;\times \;s_{ep_{m-1},m}^{†}s_{gp_{m-1},m}\;s_{gp_{m},m}^{†}s_{ep_{m},m}]\\ \;+ig_{2}[\sum_{l,m}\frac{1}{\sqrt{L}}e^{-ik^{\prime }\cdot l}\{r_{(l_{1}b\;p_{m+1};l_{2}ap_{m}),m}^{†}r_{(l_{1}c_{1}\;p_{m};l_{2}c_{1}p_{m}),m}\\ \;+r_{(l_{1}a\;p_{m};l_{2}\;bp_{m+1}),m}^{†}r_{(l_{1}c_{2}\;p_{m};l_{2}c_{2}p_{m}),m}\}s_{gp_{m-1},\;m}s_{gp_{m+1},m}^{†}]\\ \;-\;\gamma_{B,\;l}\frac{1}{L}\sum_{l}e^{-ik^{\prime }\cdot l}\;\sum_{k}{\tilde{B}}_{k}^{†}e^{ik\cdot l}+\frac{1}{\sqrt{L}}\sum_{l}e^{-ik^{\prime }\cdot l}F_{B,l}^{†}\\ \;+\;\frac{1}{L}\sum_{l}e^{-ik^{\prime }\cdot l}\sum_{l^{\prime }}J_{ll^{\prime }}\;\sum_{k}{\tilde{B}}_{k}^{†}e^{ik\cdot l^{\prime }}. \end{array}$$

The last, rather complicated term can be simplified if we introduce the center of gravity *x* = *l* + *l*′/2 and the distance *d* = *l* − *l*′. Further, it is supposed that the coupling constant *J* depends only on the distance *J*_*ll*′_ = *J*_*d*_ and the lane length L is defined by = *L*_*x*_*L*_*d*_. It follows that:

(5.50) $$\begin{array}{l} \sum_{l}\sum_{l^{\prime }}J_{d}\frac{1}{L}e^{ik\cdot l^{\prime }-ik^{\prime }\cdot l}=\;\delta_{kk^{\prime }}\frac{1}{L_{d}}\sum_{d}e^{i\left(k+k^{\prime }\right)\cdot \frac{d}{2}}J_{d}\\ \;=\;\frac{1}{L_{d}}\sum_{d}e^{ik^{\prime }\cdot d}J_{d}=\omega_{k^{\prime }} \end{array}$$

where ω_*k*_ has the dimension of a frequency. We continue with the simplification process and set

(5.51) $$\Gamma_{kk^{\prime }}=\frac{1}{L}\sum_{l}\;e^{i(k-k^{\prime })\cdot l}\;\gamma_{B,\;l}=\delta_{kk^{\prime }}\gamma_{B,}$$

if all damping constants are set equal to

(5.52) $$\gamma_{B,l}=\;\gamma_{B}.$$

(5.53) $${\tilde{F}}_{k^{\prime }}^{†}=\;\frac{1}{\sqrt{L}}\sum_{l}e^{-ik^{\prime }\cdot l}F_{l}^{†}.$$

Finally, after all these simplifications the resulting formula is obtained:

(5.54) $$\begin{array}{l} {\dot{\tilde{B}}}_{k^{\prime }}^{†}=\;ig_{1}\frac{1}{\sqrt{L}}[\sum_{l,m}\;e^{-ik^{\prime }\cdot l}\{r_{\left(l_{1}ap_{m-1};\;l_{2}\;bp_{m}\right),\;m}^{†}\;r_{(l_{1}c_{1}\;p_{m};\;l_{2}\;c_{1}p_{m}),m}\\ \;+\;r_{\left(l_{1}bl;\;l_{2}\;ap_{m-1}\right),\;m}^{†}\;r_{(l_{1}c_{2}\;p_{m};\;l_{2}\;c_{2}p_{m}),m}\}\\ \;\times \;s_{ep_{m-1},m}^{†}s_{gp_{m-1},m}\;s_{gp_{m},m}^{†}s_{ep_{m},m}]\\ \;+ig_{2}\frac{1}{\sqrt{L}}[\sum_{l,m}e^{-ik^{\prime }\cdot l}\{r_{(l_{1}bp_{m+1};l_{2}ap_{m}),m}^{†}r_{(l_{1}c_{1}\;p_{m};l_{2}\;c_{1}p_{m}),m}\\ \;+r_{(l_{1}a\;p_{m};l_{2}bp_{m+1}),m}^{†}r_{(l_{1}c_{2}p_{m};l_{2}c_{2}p_{m}),m}\}s_{gp_{m-1},\;m}\;s_{gp_{m+1},m}^{†}]\\ \;-\;\gamma \;{\tilde{B}}_{k^{\prime }}^{†}+{\tilde{F}}_{k^{\prime }}^{†}+\omega_{k^{\prime }}{\tilde{B}}_{k^{\prime }}^{†}. \end{array}$$

The basic solution to Equation (3.67) can be achieved if only the term *k*′ = 0 that corresponds to the mode with an infinite wavelength is kept. We will now solve this “reduced” equation.

(5.55) $$\begin{array}{l} {\dot{\tilde{B}}}_{0}^{†}=\;ig_{1}\frac{1}{\sqrt{L}}[\sum_{m}\;\{r_{\left(l_{1}ap_{m-1};\;l_{2}\;bp_{m}\right),\;m}^{†}\;r_{(l_{1}c_{1}\;p_{m};\;l_{2}\;c_{1}p_{m}),m}\\ \;+\;r_{\left(l_{1}bp_{m};\;l_{2}\;ap_{m-1}\right),\;m}^{†}\;r_{(l_{1}c_{2}\;p_{m};\;l_{2}\;c_{2}p_{m}),m}\}\\ \;\times s_{ep_{m-1},m}^{†}s_{gp_{m-1},m}\;s_{gp_{m},m}^{†}s_{ep_{m},m}]\\ \;+\;ig_{2}\frac{1}{\sqrt{L}}[\sum_{\;m}\{r_{(l_{1}bp_{m+1};\;l_{2}ap_{m}),m}^{†}\;r_{(l_{1}c_{1}\;p_{m};\;l_{2}\;c_{1}p_{m}),m}\\ \;+r_{(l_{1}a\;p_{m};l_{2}\;bp_{m+1}),m}^{†}r_{(l_{1}c_{2}\;p_{m};l_{2}\;c_{2}p_{m}),m}\}s_{gp_{m-1},m}\;s_{gp_{m+1},m}^{†}]\\ \;-\;\gamma {\tilde{B}}_{0}^{†}+{\tilde{F}}_{0}^{†}+\omega_{0}{\tilde{B}}_{0}^{†}. \end{array}$$

The last term, $\omega_{0}{\tilde{B}}_{0}^{†}$, can be deleted in the interaction representation and the expectation value of ${\tilde{F}}_{0}^{†}$ can be ignored. These two modifications lead to the following equation:

(5.56) $$\begin{array}{l} {\dot{\tilde{B}}}_{0}^{†}=\;ig_{1}\frac{1}{\sqrt{L}}[\sum_{m}\;\{r_{\left(l_{1}ap_{m-1};\;l_{2}\;bp_{m}\right),\;m}^{†}\;r_{(l_{1}c_{1}\;p_{m};\;l_{2}\;c_{1}p_{m}),m}\\ \;+\;r_{\left(l_{1}bp_{m};\;l_{2}\;ap_{m-1}\right),\;m}^{†}\;r_{(l_{1}c_{2}\;p_{m};\;l_{2}\;c_{2}p_{m}),m}\}\\ \;\times \;s_{ep_{m-1},m}^{†}s_{gp_{m-1},m}\;s_{gp_{m},m}^{†}s_{ep_{m},m}]\\ \;+ig_{2}\frac{1}{\sqrt{L}}[\sum_{\;m}\{r_{(l_{1}b\;p_{m+1};\;l_{2}ap_{m}),m}^{†}\;r_{(l_{1}c_{1}\;p_{m};\;l_{2}\;c_{1}p_{m}),m}\\ \;+r_{(l_{1}a\;l;\;l_{2}\;bp_{m+1}),m}^{†}\;r_{(l_{1}c_{2}\;p_{m};\;l_{2}\;c_{2}p_{m}),m}\}s_{gp_{m-1},m}\;s_{gp_{m+1},m}^{†}]\\ \;-\;\gamma {\tilde{B}}_{0}^{†}. \end{array}$$

Comparing the result of Equation (5.56) with the original expression (4.7), two main differences are immediately observable. At first, the two coupling constants, *g*_1_ and *g*_2_, have to be replaced by $g_{1}\frac{1}{\sqrt{L}}$ and $g_{2}\frac{1}{\sqrt{L}}$. Second, there is a summation over all lanes *m* = 1, …, *M*. The elaborate calculations of this subchapter show that the synchronization of motion can easily be accomplished (at least for ${\tilde{B}}_{0}^{†}$ by replacing the coupling constants and performing an addition of all lanes at the same positions.

### Running wave solutions

We again concentrate on a single robot that moves on a lane and restrict ourselves for simplicity to step 1 since the remaining steps can be handled very similarly. Furthermore, the damping constants with respect to the molecular robot are equaled: γ_*ab*_ = γ_*ba*_ = γ, the damping constants of the substrate are inoperative: γ_*g*_ = γ_*e*_ = 0, and the damping constant of the *B*-field is assumed to be effectively γ_*B*_ ≠ 0. The positions on the lane are *p*_*k*_ = *k*, *p*_*k* + 1_ = *k* + 1, … Under these assumptions, the approach that guides us to a running wave solution is formulated as follows:

(5.57) $$r_{l_{1}a\;p_{k};\;l_{2}b\;p_{k+1}}^{†}=\;R_{ab}^{†}e^{iK/(k+1)},$$

(5.58) $$r_{l_{1}c_{1}\;p_{k+1};\;l_{2}c_{1}p_{k+1}}^{†}=\;R_{c_{1}}^{†}e^{iK/(k+1)},$$

(5.59) $$s_{gp_{k}}^{†}=\;S_{g}^{†}e^{iK/k},$$

(5.60) $$s_{ep_{k}}^{†}=\;S_{e}^{†}e^{iK/k},$$

(5.61) $$s_{gp_{k+1}}^{†}=\;{\tilde{S}}_{g}^{†}e^{iK/(k+1)},$$

(5.62) $$s_{ep_{k+1}}^{†}=\;{\tilde{S}}_{e}^{†}e^{iK/(k+1)},$$

(5.63) $$B_{p_{k+1}}^{†}=\;B_{b}^{†}e^{iK/(k+1)},$$

where *K* is a real variable that will be later defined.

Insertion of these expressions into the Equations (5.4) and (5.5) provides the following formulas:

(5.64) $$\begin{array}{l} {\dot{R}}_{ab}^{†}=\;ig_{1}S_{e}S_{g}^{†}\;{\tilde{S}}_{g}{\tilde{S}}_{e}^{†}R_{c_{1}}^{†}B_{b}^{†}e^{iK/\left(k+1\right)}-\gamma R_{ab}^{†}\\ \;=\;ig_{1}D\;R_{c_{1}}^{†}B_{b}^{†}e^{iK/\left(k+1\right)}\;-\gamma R_{ab}^{†}, \end{array}$$

(5.65) $$\begin{array}{l} {\dot{R}}_{c_{1}}^{†}=\;ig_{1}S_{e}^{†}S_{g}{\tilde{S}}_{g}^{†}{\tilde{S}}_{e}R_{ab}^{†}B_{b}e^{-iK/(k+1)}-\;\gamma R_{c_{1}}^{†}\\ \;=\;ig_{1}D^{†}R_{ab}^{†}B_{b}e^{-iK/(k+1)}-\;\gamma R_{c_{1}}^{†}. \end{array}$$

In doing so, we introduced the following abbreviations:

$$D_{1}=S_{e}S_{g}^{†}=S_{g}^{†}S_{e},D_{2}={\tilde{S}}_{g}{\tilde{S}}_{e}^{†}={\tilde{S}}_{e}^{†}{\tilde{S}}_{g},D=D_{1}D_{2}=\;D_{2}D_{1}.$$

The corresponding bosonic operators (and Hermitean adjoint operators) commutate, thus they can be interchanged.

**Figure 11:**
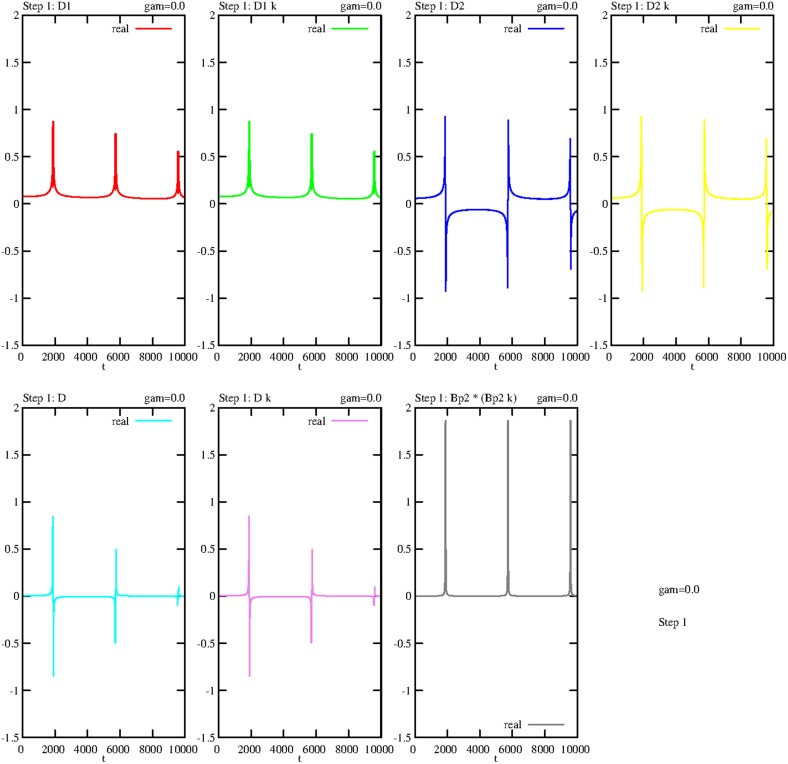
**Undamped temporal curves, showing the real parts of the operator products *D*_1_, *D*^†^_1_ (abridged to *D*_1_*k*), *D*_2_, *D*^†^_2_ (abridged to *D*_2_*k*), *D*, *D*^†^ (abridged to *D*k) and *B*^†^_*p*_2__*B*_*p*_2__, consecutively outlined for step 1**. The damping constant is γ = 0, the coupling constants are constant: *g*_1_ = *g*_2_ = 0.1.

Figure 11 shows the -like peaks of these operator products at the same time slots. At the same time, e.g., the ground state *g* will be annihilated and the exited state *e* created. The periodicity of this self-consistent process is due to its calculation without damping (revealing again the original mathematical symmetry). In a more realistic case, damping effects are activated and the curves converge after the first peak to zero. Therefore, for a fixed time *t*, both the real and imaginary parts of *D* and/or *D*^†^ are constant expressions.

In the next step, we reformulate the Equations (5.6–5.10) by including the D–terms:

(5.66) $${\dot{s}}_{g}^{†}=\;ig_{1}S_{e}^{†}D_{2}^{†}R_{ab}^{†}R_{c_{1}}B_{b}e^{-iK/(k+1)}-\gamma S_{g}^{†},$$

(5.67) $${\dot{s}}_{e}^{†}=\;ig_{1}S_{g}^{†}D_{2}R_{c_{1}}^{†}R_{ab}\;B_{b}^{†}\;e^{iK/(k+1)}-\gamma S_{e}^{†},$$

(5.68) $${\dot{\tilde{S}}}_{g}^{†}=\;ig_{1}{\tilde{S}}_{e}^{†}D_{1}R_{c_{1}}^{†}R_{ab}B_{b}^{†}e^{iK/(k+1)}-\gamma {\tilde{S}}_{g}^{†}$$

(5.69) $${\dot{\tilde{S}}}_{e}^{†}=\;ig_{1}{\tilde{S}}_{g}^{†}D_{1}^{†}R_{ab}^{†}R_{c_{1}}B_{b}e^{-iK/(k+1)}-\gamma {\tilde{S}}_{g}^{†},$$

(5.70) $${\dot{B}}_{b}^{†}=\;ig_{1}D^{†}R_{ab}^{†}R_{c_{1}}e^{-iK/(k+1)}-\gamma_{B}B_{b}^{†}.$$

The calculations are continued by substituting the three operators *R*^†^_*ab*_, *R*^†^_*c*_1__ and *B*^†^_*b*_ by

(5.71) $$R_{ab}^{†}=\rho_{ab}^{†}e^{-\gamma t},R_{c_{1}}^{†}=\rho_{c_{1}}^{†}e^{-\gamma t},B_{b}^{†}=\rho_{b}^{†}e^{-\gamma_{B}t}.$$

The derivatives of the first two expressions read as follows:

(5.72) $${\dot{R}}_{ab}^{†}={\dot{\rho }}_{ab}^{†}e^{-\gamma t}-\gamma R_{ab}^{†},{\dot{R}}_{c_{1}}^{†}={\dot{\rho }}_{c_{1}}^{†}e^{-\gamma t}-\gamma R_{c_{1}}^{†}.$$

Insertion of ${\dot{r}}_{ab}^{†}$ in (5.64) provides the expression

(5.73) $${\dot{\rho }}_{ab}^{†}=ig_{1}De^{-\gamma_{B}t}\left(\rho_{c_{1}}^{†}\rho_{b}^{†}\right)e^{iK/\left(k+1\right)}\equiv ig_{1}Ge^{-i\varphi }e^{-\gamma_{B}t}\left(\rho_{c_{1}}^{†}\rho_{b}^{†}\right),$$

where $\dot{\varphi }$ = 0 and both φ and G are real.

By inserting ${\dot{R}}_{c_{1}}^{†}$ in (5.65), the following expression is obtained:

(5.74) $${\dot{\rho }}_{c_{1}}^{†}=ig_{1}D^{†}e^{-\gamma_{B}t}\left(\rho_{ab}^{†}\rho_{b}\right)e^{-iK/(k+1)}\equiv ig_{1}\frac{G}{2}e^{i\varphi }e^{-\gamma_{B}t}\left((\rho_{ab}^{†}\rho_{b}\right).$$

It follows, by insertion of *B*^†^_*b*_, given by Equation (5.71) into expression (5.70), that

(5.75) $$\begin{array}{l} {\dot{\rho }}_{b}^{†}=\;ig_{1}D^{†}e^{-\left(2\gamma -\gamma_{B}\right)t}\left(\rho_{ab}^{†}\rho_{c_{1}}\right)e^{-iK/\left(k+1\right)}\\ \;\equiv \;ig_{1}\frac{G}{2}e^{i\varphi }e^{-(2\gamma -\gamma_{B})t}(\rho_{ab}^{†}\rho_{c_{1}}). \end{array}$$

To reproduce the r.h.s. of these three Equations (5.73–5.75), the following assumption is pursued:

(5.76) $$\rho_{ab}^{†}=\rho_{0}e^{F(t)},\rho_{c_{1}}^{†}=\rho_{0}e^{\frac{F(t)}{2}}e^{-i\varphi },\rho_{b}^{†}=\rho_{0}e^{\frac{F(t)}{2}}e^{2i\varphi },$$

where $F\left(t\right)=\int_{0}^{t}G\left(\tau \right)d\tau ,\;\rho_{0}=\text{const}.\in \mathbb{R}$.

With this ansatz the derivatives of the three operators ρ^†^_*ab*_, ρ^†^_*c*_1__, ρ^†^_*b*_ can be cast in the following form:

(5.77) $${\dot{\rho }}_{ab}^{†}=iG\rho_{ab}^{†}=iG\frac{1}{\rho_{0}}\left(\rho_{c_{1}}^{†}\rho_{b}^{†}\right)e^{-i\varphi },$$

(5.78) $${\dot{\rho }}_{c_{1}}^{†}=i\frac{G}{2}\rho_{c_{1}}^{†}=i\frac{G}{2}\;\frac{1}{\rho_{0}}\left(\rho_{ab}^{†}\rho_{b}\right)e^{i\varphi },$$

(5.79) $${\dot{\rho }}_{b}^{†}=i\frac{G}{2}\;\rho_{b}^{†}=i\frac{G}{2}\frac{1}{\rho_{0}}\left(\rho_{ab}^{†}\rho_{c_{1}}\right)e^{i\varphi }.$$

From expression (5.83), the value of *G* is concluded as:

(5.80) $$\rho_{0}Re\left(D\right)e^{iK/\left(k+1\right)}e^{i\varphi }=G.$$

Both sides of this expression should be real, therefore the exponents must be zero: φ = − *K*/(*k* + 1) and ρ_0_Re(*D*) = *G*. A second solution is formed by the same φ but with $\rho_{0}\text{Re}\left(D\right)=\frac{G}{2}$. This last result comes from the two Equations (5.74) and (5.75).

Due to the determination of the two variables φ and *K*, running wave solutions of the molecular “leg-over-leg” walking can be expressed for the three operators $r_{l_{1}a\;p_{1};\;l_{2}b\;p_{2}}^{†},\;r_{l_{1}c_{1}\;p_{2};\;l_{2}c_{1}p_{2}}^{†}$, *B*^†^_*p*_2__ in the conventional form (*k* = 1, 2,..; *G* = ρ_0_*Re*(*D*) = const.):

(5.81) $$r_{l_{1}a\;p_{k};\;l_{2}b\;p_{k+1}}^{†}=\;e^{i\left(Gt-\varphi \left(k+1\right)\right)}\;\rho_{0}e^{-\gamma t},$$

(5.82) $$r_{l_{1}c_{1}\;p_{k+1};\;l_{2}c_{1}p_{k+1}}^{†}=\;e^{i\left(Gt-\varphi \left(k+1\right)\right)}\;\rho_{0}e^{-\gamma t},$$

(5.83) $$B_{p_{k+1}}^{†}=e^{i\left(Gt-\varphi \left(k+1\right)\right)}\;\rho_{0}e^{-\gamma_{B}t}.$$

Such a solution is not surprising because it is well known that a composition of different modes (more than only the ground mode *k* = 0) can generate running waves (see Section Coherent Motion on Parallel Substrate Lanes).

## Discussion

The central goal of this contribution was to support the hypothesis that quantum mechanical effects in molecular biology—especially in human brains—cannot be neglected. This assumption is mainly justified by the size of the interacting objects (nano size) and by the experimental results showing that even greater molecules up to about 100 atoms can demonstrate quantum behavior, e.g., in double-slit experiments (Haken and Levi, 2012). The nano size argument is further supported by the established experimental technology to construct molecular machines and walkers from DNA.

The most striking results to emerge from the produced solutions is that neural processes like the motion of molecular robots (kinesin and dynein) in axons and dendrites can be modeled by an approach that is particle-based (impact solution) or purely wave-oriented (synchronized modes solution and running wave solution). In addition, wave-based solutions also seem to be very relevant for the interactions between different neural layers.

We are aware that our predictions suffer from limitations and have to be considered as a first, basic approach due to the following three reasons. Firstly, the number of the many parameters (in total 16) was reduced to four (two coupling constants and two damping constants). Secondly, there are still not enough experimental data to fix the values of the last mentioned four parameters or even the great set of all relevant available biological data. Thirdly, a greater number of experimentally approved parameters could lead to a higher generalization of the achieved results.

A first extension of our approach could be the integration of tunneling effects, the inclusion of fluctuation forces, and the consideration of the cargo transport. All these additional considerations would greatly increase the predictive power of the outlined model. In this way, it would be feasible to describe e.g., the walk of a molecular robot along the DNA and the transcription of a nucleotide into RNA, whereby for each step the effective potential barrier must be tunneled.

### Conflict of interest statement

The author declares that the research was conducted in the absence of any commercial or financial relationships that could be construed as a potential conflict of interest.
